# The influence of weather on mortality in rural Tanzania: a time-series analysis 1999–2010

**DOI:** 10.3402/gha.v5i0.19068

**Published:** 2012-11-23

**Authors:** Sigilbert Mrema, Amri Shamte, Majige Selemani, Honorati Masanja

**Affiliations:** Ifakara Health Institute, Coordination Office, Kiko Avenue, Mikocheni, Dar es Salaam, Tanzania

**Keywords:** time-series, monthly weather, all-cause mortality, monthly temperature and monthly average temperature climate, climate change

## Abstract

**Background:**

Weather and climate changes are associated with a number of immediate and long-term impacts on human health that occur directly or indirectly, through mediating variables. Few studies to date have established the empirical relationship between monthly weather and mortality in sub-Saharan Africa.

**Objectives:**

The objectives of this study were to assess the association between monthly weather (temperature and rainfall) on all-cause mortality by age in Rufiji, Tanzania, and to determine the differential susceptibility by age groups.

**Methods:**

We used mortality data from Rufiji Health and Demographic Surveillance System (RHDSS) for the period 1999 to 2010. Time-series Poisson regression models were used to estimate the association between monthly weather and mortality adjusted for long-term trends. We used a distributed lag model to estimate the delayed association of monthly weather on mortality. We stratified the analyses per age group to assess susceptibility.

**Results:**

In general, rainfall was found to have a stronger association in the age group 0–4 years (RR=1.001, 95% CI=0.961–1.041) in both short and long lag times, with an overall increase of 1.4% in mortality risk for a 10 mm rise in rainfall. On the other hand, monthly average temperature had a stronger association with death in all ages while mortality increased with falling monthly temperature. The association per age group was estimated as: age group 0–4 (RR=0.934, 95% CI=0.894–0.974), age group 5–59 (RR=0.956, 95% CI=0.928–0.985) and age group over 60 (RR=0.946, 95% CI=0.912–0.979). The age group 5–59 experienced more delayed lag associations. This suggests that children and older adults are most sensitive to weather related mortality.

**Conclusion:**

These results suggest that an early alert system based on monthly weather information may be useful for disease control management, to reduce and prevent fatal effects related to weather and monthly weather.

Weather and climate changes are likely to have adverse effects on human health, particularly among the most vulnerable populations (1). Their effects can be both direct, through extreme events and changes in the disease environment, and indirect through their impact on the economic livelihood of the population (1, 2). The World Health Organization (WHO) estimated that the warming and rainfall trends due to anthropogenic climate change of the past 30 years already claim over 150,000 lives annually (3). In most cases the poorer regions are highly vulnerable (4). Of the 14 million deaths that occurred in Southeast Asia annually, 40% are attributable to communicable diseases (5). Increased average temperatures could prolong peak periods for vector-borne diseases (6) and extreme weather events such as cyclones and floods can create ideal conditions for the spread of vector-borne and diarrheal diseases such as cholera (7). Malaria is the most important vector-borne disease related to climate change in the world; it is also a preventable disease. About 40% of the world's population is at risk of contracting malaria, and roughly 75% of cases occur in Africa, with the remainder occurring in Southeast Asia, the western Pacific, and the Americas (8). In sub-Saharan Africa, malaria remains the most common parasitic disease and is the main cause of morbidity and mortality among children less than 5 years of age, elderly people, and among pregnant women (9). The estimates provided by Murray and Lopez (1996) suggested that malaria caused about 15% of deaths of children under the age of 5 years in sub-Saharan Africa in 1990. However, children are highly affected by weather and climate by virtue of their early stages of development, while a study done in Kenya in 2008, revealed that climate change has emerged as a new driver of malnutrition and increasing the child mortality rate by 5–20 times (10).

Tanzania is the largest country in East Africa, covering an area of 945,200 km^2^, 60,000 km^2^ of which is inland water (11). Tanzania lies close to the equator on the east coast of Africa between latitude 1°S and 12°S and longitude 30°E and 40°E. By being close to the equator, the climate variations in temperature are not very extreme (11). Changes in temperature and rainfall resulting in changes in soil moisture, increase in sea level, and more extreme weather events, such as floods and droughts, are among the most known impacts of global climate change in the region (12, 13). The major impacts of climate change are expected to include severe floods, frequent and prolonged droughts, rising sea levels, crop failure, loss of livestock, lower water availability and quality, and an increase in vector and water-borne diseases (14, 15). However, heavy rains, flood, drought, and landslides in Tanzania have already resulted into internal displacement, food shortages, and increased disease transmissions. Drought itself has significantly contributed to malnutrition due to lack of adequate food, increased infectious disease transmission, and scarcity of clean and safe water (16). Landslides, droughts, and floods are becoming common in Tanzania. In recent years (2009–2011), heavy rains accompanied with strong winds have left thousands of people displaced and without food in Muleba, Kilosa, Same, and Dar es Salaam (17). Weather and climate change can affect human health and well-being through a variety of mechanisms (14, 15, 18). The risk of emerging diseases may increase due to changes and survival of pathogens in the environment, changes in migration pathways, carriers and vectors, and changes in the natural ecosystems (18). Infectious agents are in a state of perpetual adaptation to their new host(s) or vectors which can lead to the emergence of ‘new’ disease(s) or the spread of known diseases in previously unaffected areas. Malaria is by far the most important vector-borne disease causing high morbidity and mortality in Tanzania (19). The endemicity and pattern of malaria transmission is focal and varies from place to place depending on many factors including weather and topography. Modeling malaria endemicity in Tanzania using outpatient cases (2004–2008) in relation to mean temperature and mean rainfall has shown that almost the whole of Tanzania is endemic for malaria, although with some spatial variation between areas. Mean rainfall accounts for 72% of the variation in malaria, while mean maximum temperature accounts for 14.1% and mean minimum temperature 13.1% (19).

The main objective of this paper is to show a detailed analysis of the association between changes of monthly weather (temperature and rainfall) on all-cause mortality by age groups, using the data from Rufiji Health and Demographic Surveillance System (RHDSS) and metrological data of rainfall and temperature for the period of 1999–2010.

## Methods

### Study area

**Fig. 1:**
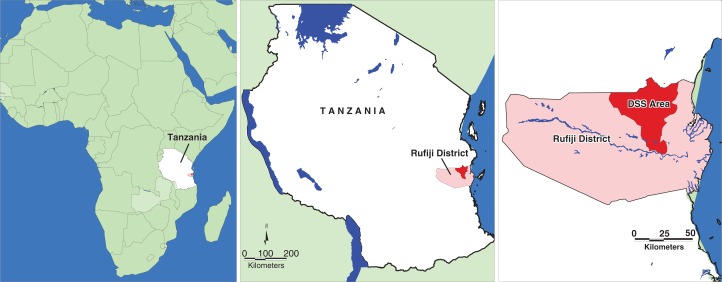
Location of Rufiji HDSS.

The RHDSS is located in eastern Tanzania 7.47° to 8.03° south latitude and 38.62° to 39.17° east longitude (Fig. 1). The RHDSS is in the Rufiji district of Tanzania about 178 km south of Dar es Salaam. The district is among the six districts in the Coastal Region of Tanzania. The RHDSS constitutes 31 villages covering an area of 1813 km^2^ (20, 21).

Rufiji has a mean altitude of <500 meters above sea level. Tropical forests and grassland dominated the vegetation cover of Rufiji. The weather is hot all over the year and with rainy seasons: short rains (October–December) and long rains (February–May). The average annual rainfall in the district is between 800 and 1,000 mm (21). The population size of the Rufiji District is about 203,102 of which more than 85,000 (about 42% of the district population) are covered by the surveillance system (22). The mean household size for the whole district is about five people per house (22). The district is largely rural and the population is clustered in small townships in the district (21).

RHDSS has a total of 18 health facilities. These include one hospital, two health centers, and 15 dispensaries. However, there are a proportion of people who receive health services from traditional healers and traditional birth attendants. The major causes of mortality include acute febrile illness such as malaria, AIDS, acute lower respiratory infections, tuberculosis, and perinatal causes. Immunization coverage ranges from 66% for measles in children that are 12–23 months of age to 85% for the Bacillus Calmette-Gue'rin (BCG; tuberculosis). About 89% of the population lives within 5 km of a formal health facility. All villages and health facilities in the district have been positioned by a global position system and mapped in a geographic information system database of the district health resources.

### Data and study population

This study used longitudinal data collected in the RHDSS over an 11-year period from 1 January 1999 to 31 December 2010. A DSS is a longitudinal, population-based, health and vital events registration system that monitors demographic events such as births, deaths, pregnancies, migrations and socioeconomic status of a geographically well-defined setting of individuals, households, and residential units. In the RHDSS, every household is visited once in every 4 months in order to update previously recorded household information, which also includes registering new demographic events that may have occurred. Between household visits, community-based key informants report births and deaths as they occur, and when it does, a household is revisited in order to record such events.

Since the main focus of this study was to analyze the association of rainfall and temperature on all-cause mortality by age; all deaths that occurred and were recorded during the study period were of interest. Data on monthly rainfall (mm) and temperature (°C) were obtained from the Tanzania Meteorological Authority (TMA) head office in Dar es Salaam. TMA provides meteorological services, weather forecasts, climate services, and warnings including daily forecast information for each region in Tanzania (23).

## Statistical analysis

Data management was done by using Intercooled Stata 11 (24) and analysis was performed using the MGCV package in R2.14.2 is a system for statistical computation and graphics. It consists of a language plus a run-time envirnment with graphics, a debugger, access tocertain system functions, and the ability to run programs sorted to script files (25). The MGCV package provides tools for generalized additive models (GAMs) and other generalized ridge regression (26). GAMs have been widely used in many time-series analyses and have been effectively applied in a variety of research areas (27). Data process was performed to generate the variables for time-series analysis as follows: all-causes mortality from the year 1999 to 2010; data on rainfall; and data for temperature were reported in mean, maximum and minimum. Age at death was grouped into three age groups: 0–4, 5–59, and over 60 years. A division was made between male and female. Season, time, and lags (lag 0–4) for both monthly rainfall and average monthly temperature were generated as well. Time-series Poisson regression models, using the MGCV package in this study, account for autocorrelation, seasonality, long-term trends, and lag effects that determine the best-fit model in relation to all-cause mortality attributed by monthly rainfall and monthly average temperature by age. The long-term trend was modeled through a natural cubic spline curve with 3 degrees of freedom per year of data. The degree of freedom for each smooth term in the model are chosen simultaneously as part of model fitting by minimizing the generalized cross-validation score of the whole model (26). The annual seasonal variation was modeled through natural cubic splines with 3 degrees of freedom also. Thus, the combined degree of freedom for both season and trend add up to 6 degrees of freedom per year. Four lags for rainfall and temperature were created in order to assess the delayed association of the previuos rainfall and temperature values on the current level of mortality. The GAM model used in this analysis was given by:

$$Y_{t}\sim \text{Poisson}({µ}_{t})$$

$$\log({µ}_{t})=\alpha +\sum_{i=1}^{12}s(x_{it},df)+s({\text{time}}_{t},df)$$

Where *t* refers to the month of the observation; (*Y*_*t*_) denotes the observed monthly mortality counts on month *t*; *s*(.) denotes a smooth cubic spline function, *df* denotes degrees of freedom, *xi* denotes the monthly rainfall at lag 0–4 and monthly average temperature at lag 0–4, and ‘time’ represents both seasonal and trend pattern. We quantified the associations between the weather variables and mortality by strata of age, and the equation above was fitted for each age group.

### Ethical approval

The ethical clearance for RHDSS was approved by local and national ethical committees.

## Results

### Description

Table 1 represents the summary of all deaths recorded in the RHDSS aggregated per month from 1999 to 2010. A total of 10,116 deaths over the 11 years of observation (1999–2010) were recorded. The percentages of total deaths by age group were 30% for 0–4 years, 7% for 5–19 years, 23% for 20–59 years, and 40% for over 60 years. Since the proportion of age group 5–19 was very small we merged it with the age group 20–59 for further analysis. According to the RHDSS burden of disease profiles of 1999 to 2010, the large proportion of all-causes of deaths in the region were from communicable diseases. Malaria, HIV/AIDS, TB, and pneumonia are the leading cause of mortality for all age groups followed by a variety of neonatal and under-fives’ problems such as low birth weight and birth asphyxia.

**Table 1:** Summary of monthly mortality data, 1999–2010

		All cause, by age			
			
Months	U5 (0–4)	Children (5–19)	Adults (20–59)	Adults (60+)	Total
January	276	54	177	325	832
February	290	55	216	318	879
March	201	42	172	264	679
April	325	63	224	346	958
May	317	57	225	336	935
June	315	79	236	370	1,000
July	228	43	173	319	763
August	228	65	205	406	904
September	237	73	195	377	882
October	216	73	207	355	851
November	167	45	132	248	592
December	223	64	200	354	841
Total	3,023 (30%)	713 (7%)	2,362 (23%)	4,018 (40%)	10,116

### Seasonal variability of weather and mortality

**Fig. 2:**
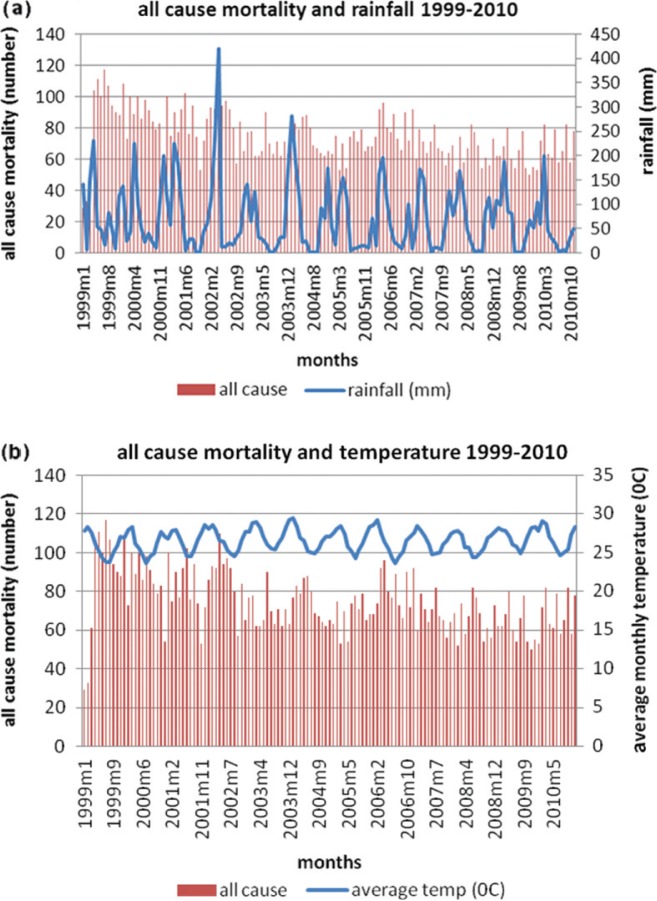
(a) Time series of all-cause mortality and rainfall; (b) Time series of all-cause mortality and temperature.

Since Rufiji has a tropical environment the monthly average temperature varied between 27.91°C and 34.4°C with a mean of 31.22°C. Monthly rainfall varied between 0 and 420.7 mm during the study period. Fig. 2a and 2b present the seasonal variations in all-cause mortality and rainfall and all-cause mortality and temperature variables, respectively, during the study period. Generally, there are seasonal fluctuations in mortality, with the highest peaks of deaths occurring during the periods of relative cold and high levels of rainfall.

### Seasonality of mortality

**Fig. 3:**
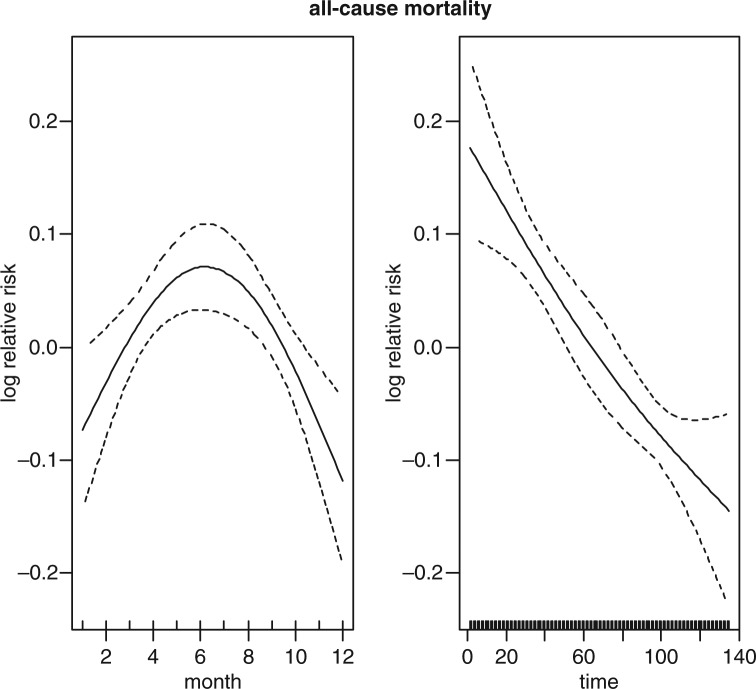
Seasonality (left) and time trends (right) in all-cause mortality over the study period.

**Fig. 4:**
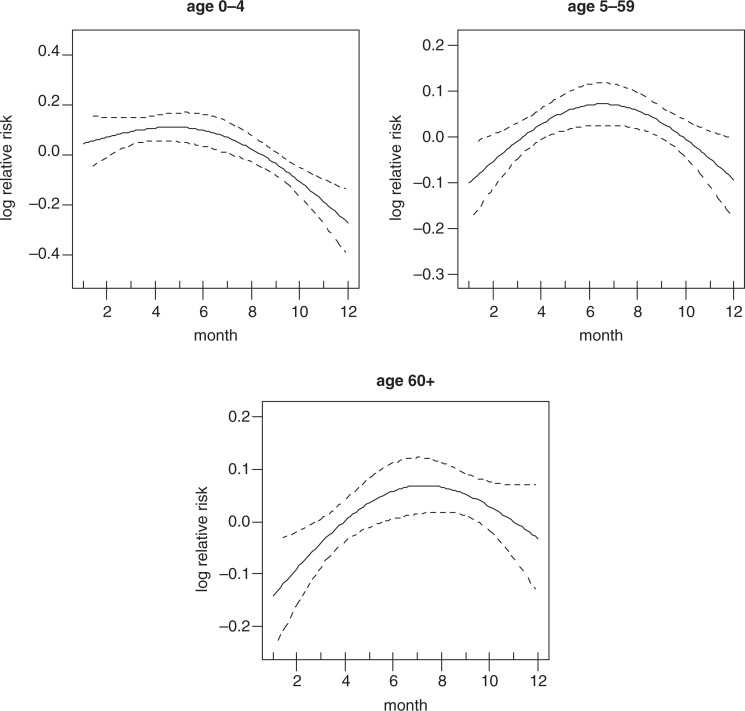
Seasonality of all-cause mortality by age group.

**Fig. 5:**
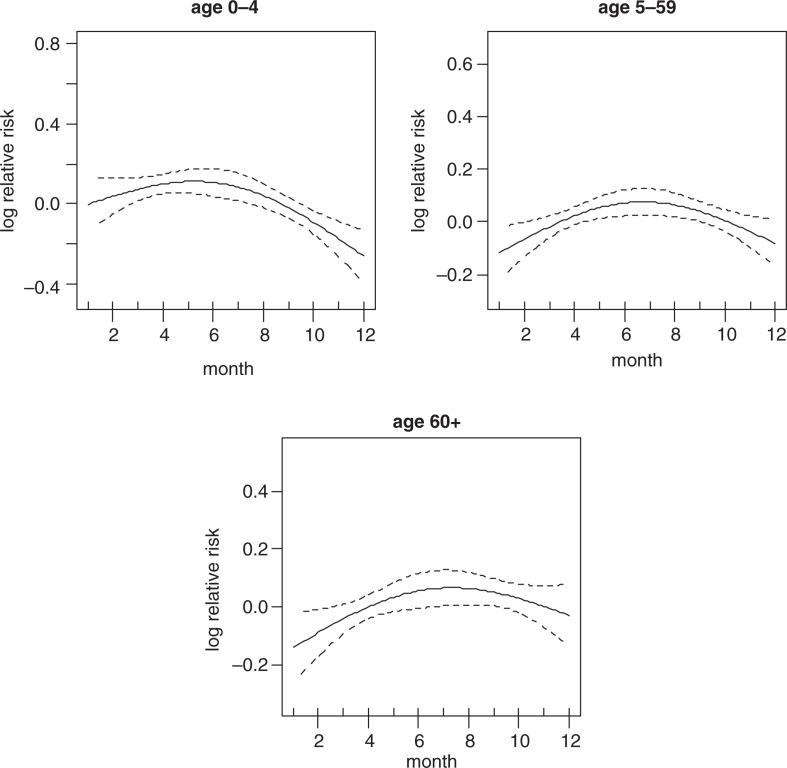
Seasonality of all-cause mortality by age group adjusted for rainfall.

Fig. 3 shows the strong crude seasonality and long-term trend of all-cause mortality. It shows that most of the deaths are concentrated in the middle of the year and also that there is a downward trend of mortality over time. The crude seasonality of all-cause mortality by specific age groups is shown in Fig. 4. A strong mortality pattern is observed in all age groups, that is, 0–4, 5–59, and over 60. High mortality is observed during March–May for the age group 0–4 and 5–59, the period that coincides with the long rain season. For the over 60 age group, mortality peaks during the months of June–September which corresponds with the dry and cold temperature period. Fig. 5 shows the adjusted seasonality of all-cause mortality for monthly rainfall by age group. It shows that for all age groups (0–4, 5–59, and over 60), the mortality pattern peaks during the period of rain. The estimated curve of seasonality adjusted for weather shows a similar pattern in children under five, but a less clear seasonal pattern in the older age group indicating that weather variables explain part of the seasonal pattern for these age groups.

**Fig. 6:**
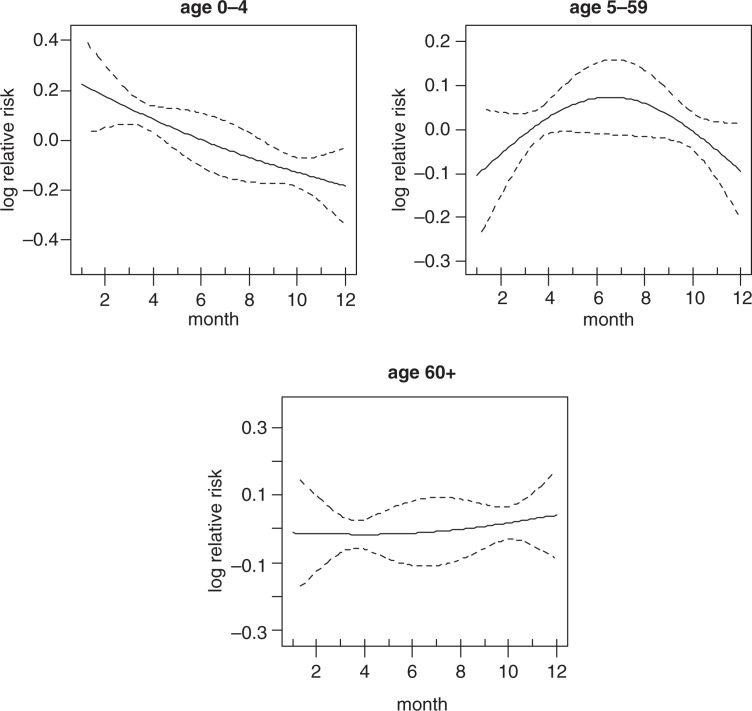
Seasonality of all-cause mortality by age group adjusted for temperature.

The adjusted seasonality of all cause mortality for monthly temperature by age group are presented in Fig. 6. The patterns show that mortality in all age groups peaked up at the mid of the year. This correspond with the time when the temperature is relatively lower compared to other periods of the year in Rufiji.

### Temperature and rainfall mortality plots

**Fig. 7:**
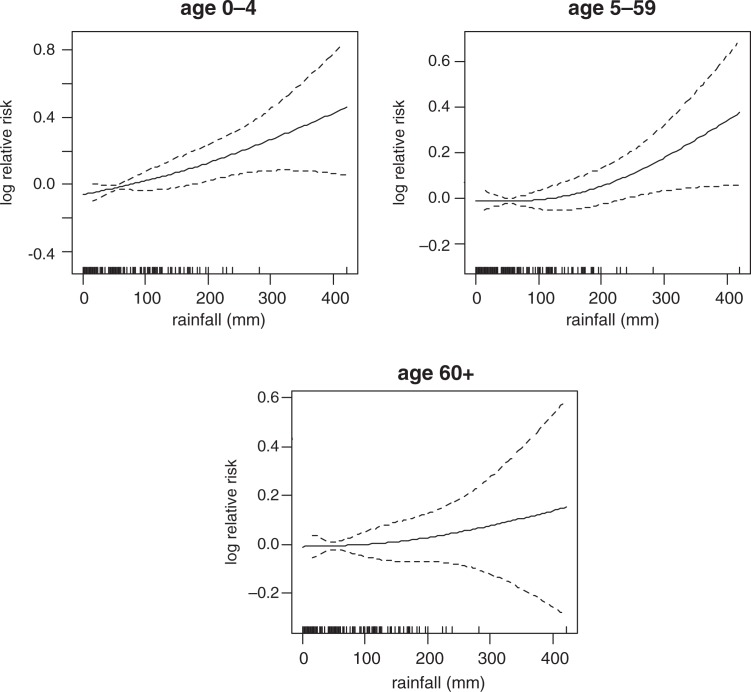
The influence of rainfall on mortality by age group.

In Fig. 7, the graphs show smoothed plots of logged relative risk of mortality against monthly rainfall. It shows a positive linear rainfall-mortality relationship in all age groups. Based on the plots, only the slope of age group 0–4 is statistically significant (RR=1.001, 95% CI=0.961–1.041) whereby an increase of 10 mm of rainfall will increase mortality in the age group 0–4 by 1.4% (Table 2). Correspondingly, if the monthly rainfall rises to 400 mm, it will correspond with a 72% increase in under-five mortality.

**Table 2:** Correlations between all-cause mortality and climate variables in RHDSS

Age group	Monthly climate variables	Coefficient	RR	95% CI	N
0–4	Monthly rainfall	0.0013662	1.001	(0.961, 1.041)	135
5–59	Monthly rainfall	0.0005424	1.001	(0.999, 1.001)	
60	Monthly rainfall	0.0001606	1.000	(0.999, 1.001)	
0–4	Monthly average temp	−0.0683968	0.934	(0.894, 0.974)	135
5–59	Monthly average temp	−0.0446006	0.956	(0.928, 0.985)	
60	Monthly average temp	−0.0560136	0.946	(0.912, 0.979)	

**Fig. 8:**
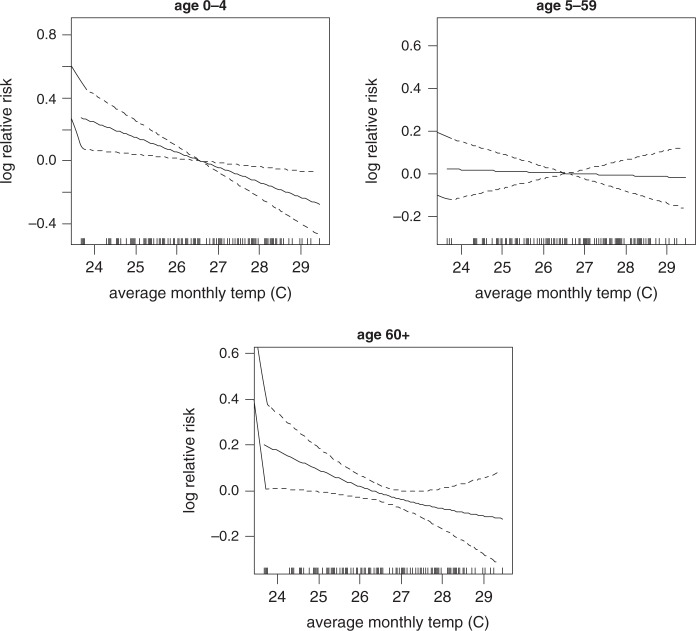
The influence of temperature on mortality by age group.

Fig. 8 shows linear plots of logged relative risk of mortality against the monthly average temperature. The graph reveals linear temperature-mortality relationships over lags. In Fig. 8, the threshold temperature exists somewhere between 26°C and 27°C for all age groups. Negative associations were observed for the temperature range below the thresholds. The monthly average temperature was significantly associated with all-cause mortality in all age groups (Table 2). If the monthly average temperature will decrease up to 24°C from the threshold, mortality will increase by 80.7%, 65.7% and 74% in age groups 0–4, 5–59, and over 60, respectively. The linear function of monthly rainfall effects on all-cause mortality shows a linear increase significant at lag of 0, 1, 2, and 3 in age group 0–4 (Table 3), while the effects of monthly average temperature on all-cause mortality is significant at lag of 2 in age group 5–59. There are no strong lag effects in the association of climatic variables (monthly rainfall and average temperature) on mortality in the age group 60 years and above (Table 3).

**Table 3:** Lag correlations between all-cause mortality and climate variables in Rufiji DSS, 1999–2010

Age Group	Monthly climate variables	Lag (months)	Coefficient	RR	95% CI	N
0–4	Monthly rainfall	0	0.000791	1.001	(1.000, 1.001)	98
	Monthly rainfall	1	0.00107	1.001	(1.000, 1.002)	
	Monthly rainfall	2	0.000893	1.001	(1.000, 1.002)	
	Monthly rainfall	3	0.000863	1.001	(1.000, 1.002)	
	Monthly rainfall	4	−0.0000627	0.999	(0.999, 1.001)	
	Monthly average temperature	0	−0.01711	0.964	(0.866, 1.062)	
	Monthly average temperature	1	−0.01111	0.995	(0.857, 1.124)	
	Monthly average temperature	2	0.071511	1.074	(0.941, 1.208)	
	Monthly average temperature	3	0.064105	1.078	(0.949, 1.207)	
	Monthly average temperature	4	−0.00736	0.969	(0.874, 1.066)	
5–59	Monthly rainfall	0	0.0004944	1.001	(0.999, 1.001)	98
	Monthly rainfall	1	−0.0001282	0.999	(0.999, 1.000)	
	Monthly rainfall	2	0.0003587	1.000	(0.999, 1.001)	
	Monthly rainfall	3	0.0003253	1.000	(0.999, 1.000)	
	Monthly rainfall	4	0.0002049	1.000	(0.999, 1.000)	
	Monthly average temperature	0	−0.009272	0.991	(0.920, 1.062)	
	Monthly average temperature	1	0.06344	1.066	(0.971, 1.161)	
	Monthly average temperature	2	−0.108909	0.897	(0.801, 0.993)	
	Monthly average temperature	3	0.080517	1.084	(0.991, 1.176)	
	Monthly average temperature	4	0.020678	1.021	(0.952, 1.089)	
60	Monthly rainfall	0	−0.0000986	0.999	(0.999, 1.001)	98
	Monthly rainfall	1	−0.000259	0.999	(0.999, 1.001)	
	Monthly rainfall	2	−0.000509	0.999	(0.999, 1.000)	
	Monthly rainfall	3	0.000454	1.000	(0.999, 1.001)	
	Monthly rainfall	4	0.000447	1.001	(0.999, 1.001)	
	Monthly average temperature	0	−0.04728	0.953	(0.857, 1.048)	
	Monthly average temperature	1	−0.00626	0.995	(0.867, 1.122)	
	Monthly average temperature	2	−0.0267	0.974	(0.845, 1.103)	
	Monthly average temperature	3	0.038513	1.039	(0.915, 1.163)	
	Monthly average temperature	4	−0.03235	0.967	(0.875, 1.060)	

## Discussion

The main focus of this study was to investigate the influence of rainfall and temperature on all-cause mortality patterns in Rufiji, Tanzania. The findings show, in particular, an association between rainfall and mortality in children. This finding was similar to that observed by other studies (17). The observed rainfall association can be substantiated by the location of Rufiji where it experiences a tropical climate with a long rainy season from February to May and short a rainy season from October to December. This pattern is consistent with deaths caused by malaria, which also peak in the long and short rains, and is the single largest disease component contributing to the burden of disease in all ages in Rufiji (28). The findings of this study are similar to previous assessments of weather related mortality (29–31). Recent studies conducted in Africa revealed that the outbreak of cholera and malaria support the causal link between monthly weather and health (32). Rainfall anomalies are widely considered to be a major driver of inter-annual variability of malaria incidence in Africa. About 90% of the deaths occurred in sub-Saharan Africa are believed to be due to malaria (12). Studies show that rainfall excess is correlated with changes in malaria incidence in certain eco-epidemiologic settings, apparently as a result of its impact on the population dynamics of the *Anopheles* spp. mosquito vector (29, 33). Weather shocks raise exposure to malaria as shown by the significant rise in the incidences of infant death (34). This is similar to our findings where rainfall is significantly associated with mortality in under-fives. The effect of rainfall on mortality can last for days, with the greatest association sometimes observed in the same month (30), which is similar to results in this study.

The association between ambient temperature and daily mortality has been well documented in the developed countries of the Northern Hemisphere (35). This study shows a relationship between both hot and cold temperature associations on mortality, although cold temperatures had a stronger association with mortality. Generally, Rufiji did not experience any extreme cold events that could directly cause mortality like that in the Northern Hemisphere countries. However, Rufiji's population is accustomed to a tropical climate and, like any other population, is exposed to cold temperatures relative to its average climate. This often occurs during the rainy season (which is consistent with the high malaria transmission season). Also it has been suggested that increased mortality is connected with cold weather because of elevated occurrences of influenza and other respiratory infections (36). Other studies show that housing factors and the substantial numbers of elderly living in ‘fuel’ poverty may influence the risk of excess winter deaths (31, 37), which is similar to results in this study. Inadequate indoor heating and outdoor clothing are likely to be important factors, therefore, in the observed associations with social deprivation. The effect of cold temperatures on mortality can be observed in the same month and can continue to last for months, as also observed in our investigation.

In conclusion, monthly weather showed a strong association on all-cause mortality. Younger age groups and the elderly population are more susceptible to the influence of monthly weather. Results of the present study show similarities with some previous findings, but also contradict other findings. The conflicting findings are mainly attributable to the multiple weather variables included in a range of studies, and to the different underlying disease burdens in studies from developed settings. This study highlights that the influence of monthly weather on all-cause mortality/morbidity is not well understood, and needs to be further studied in order to better comprehend causal mechanisms and to develop preventive actions.

This study has some limitations. For example, the analyses are based on all-cause mortality, but since the causes of death are driven by different mechanisms they also respond differently to monthly weather.

Finally, with a better understanding of health responses to weather conditions then better health services and policies could be formulated, such as stressful weather episode warnings to susceptible populations and the proper allocation of limited resources.

## Authors’ contributions

SM wrote the manuscript first draft, AS and MS revised the paper and contributed to the discussion. SM, AS, and MS analyzed data, revised the manuscript, and contributed to the discussion. HM participated in designing the study and critically reviewed the manuscript drafts. All authors read and approved the final manuscript.
